# Climate service driven adaptation may alleviate the impacts of climate change in agriculture

**DOI:** 10.1038/s42003-022-04189-9

**Published:** 2022-11-12

**Authors:** Andrea Toreti, Simona Bassu, Senthold Asseng, Matteo Zampieri, Andrej Ceglar, Conxita Royo

**Affiliations:** 1 European Commission, Joint Research Centre, Ispra, Italy; 2 Technical University of Munich, Freising, Germany; 3 IRTA, Lleida, Spain; 4 Present Address: King Abdullah University of Science and Technology, Thuwal, Kingdom of Saudi Arabia; 5 Present Address: Climate Change Centre, European Central Bank, Frankfurt, Germany

**Keywords:** Plant sciences, Agriculture

## Abstract

Building a resilient and sustainable agricultural sector requires the development and implementation of tailored climate change adaptation strategies. By focusing on durum wheat (*Triticum turgidum* subsp. *durum*) in the Euro-Mediterranean region, we estimate the benefits of adapting through seasonal cultivar-selection supported by an idealised agro-climate service based on seasonal climate forecasts. The cost of inaction in terms of mean yield losses, in 2021–2040, ranges from −7.8% to −5.8% associated with a 7% to 12% increase in interannual variability. Supporting cultivar choices at local scale may alleviate these impacts and even turn them into gains, from 0.4% to 5.3%, as soon as the performance of the agro-climate service increases. However, adaptation advantages on mean yield may come with doubling the estimated increase in the interannual yield variability.

## Abstract (web summary)

Climate modelling indicates that ecosystem services could alleviate climate change impacts on wheat yield in the Mediterranean.

## Introduction

Crop productivity may be severely affected by climate change^1,2^. As shown by regional climate change impact assessments for Europe, north-south diverging changes may already emerge at 1.5 °C warming scenarios, with (for some crops) projected yield gains in the north and losses in the Mediterranean region^2^. Despite market spillover effects that may reduce (in certain cases compensate for) the negative impacts of climate change, and wheat not being the most affected crop^1,2^, high risk remains^3^ especially associated with climate variability and extremes^4,5^.

Developing and testing effective adaptation strategies is of primary importance to enhance the climate resilience of the agricultural sector. Back in 1992, Rosenberg^6^ was already advocating climate change adaptation in agriculture, calling for conjectural assessments due to knowledge and modelling gaps. Today, assessing impacts and designing adaptation strategies^7^ can rely on ensembles of improved process-based crop models’ simulations, although limitations^8,9^ and uncertainties^10^ still remain. Among them, quantifying the added value of climate services^11^ is still a challenging task^12^, despite their role in adapting to climate change. Barriers to a comprehensive evaluation of agro-climate services are represented by the lack of fully integrated models and computational-related limitations, that may be both overcome, e.g., by recently launched Earth’s Digital Twin initiatives^13^.

In this study, we focus on the effectiveness of tailored climate services informing about optimal variety choice at sowing for durum wheat production in the Euro-Mediterranean region. Previous studies^14–17^ have focused on understanding and characterising the impacts of climate change on wheat. Here, for the first time (as far as we know) we use climate projections to simulate an idealised sectoral and tailored climate service, and quantify its benefits on wheat productivity under near-future climate conditions. The Mediterranean region is the most important durum wheat producer (mostly under rain-fed condition) and consumer^18^, due its use to making pasta, couscous, and bulgur. During the 20th century, durum wheat varieties have evolved towards higher harvest index and a larger number of grains per unit area, and lower plant height^19^; although progress has slowed down since 1980.

During a user scoping workshop of the EU-H2020 MedGOLD project (involving durum wheat farmers, breeders, and regional stakeholders; deliverables 4.6 and 4.7 available at www.med-gold.eu), variety selection at sowing was identified among the most important decisions to be supported by sectoral climate services. Based on observations, seasonal climate predictions, local information, and phenological modelling, such service can provide risk estimation and advice farmers on the cultivars minimising the risk for the growing season ahead^20,21^.

By focusing at the near future projections (2021–2040, under the high-end RCP8-5 emission scenario), we here explore the effectiveness of informed variety-selection at sowing as offered by *idealised* agro-climate services. We evaluate the benefits of such a service in reducing the negative effects of climate change on mean wheat yield and stability in the Euro-Mediterranean region. Other adaptation mechanisms, e.g., modifying different agro-management practices^22^ (e.g., sowing date, water use, fertilisation), are here not considered to avoid the impact of too many confounding factors. The idealised climate service is realised by applying a certain probability of success (dependent on an a priori-set prediction skill) to the known *reality* given by the climate projections and the crop growth model output based on such climate information (see Methods).

## Results

**Fig. 1:**
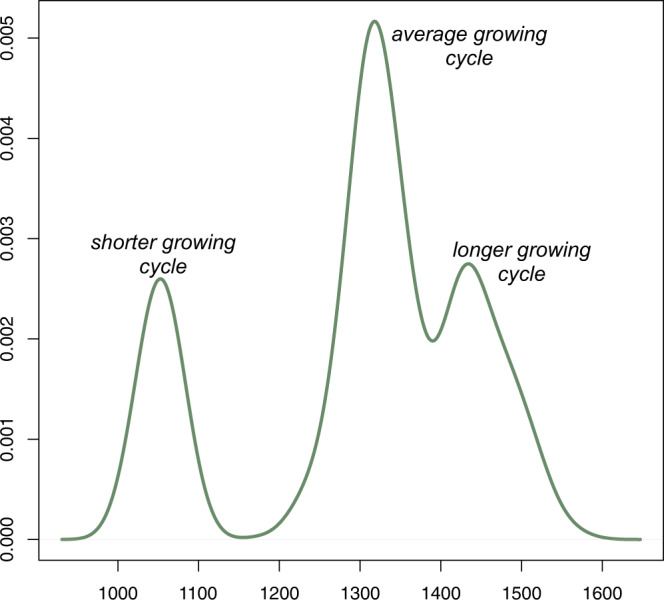
Probability density function of the thermal demand to reach anthesis. Data from the 191 accessions^23^.

To take into account the genetic diversity offered by varieties currently grown in the Euro-Mediterranean region, durum wheat accessions^23^ were analysed. Three families were identified (Fig. 1) and used to define 18 ideotypes (see Methods). Crop yields were then simulated under historical and projected climate conditions (see Methods). These ideotypes provide a reasonably good representation of durum wheat varieties to which a farmer may have access. Thus, they are here used to assess the benefits of targeted climate services informing each year on optimal (with respect to the expected climate conditions along the growing season ahead) varieties to be sown.

**Fig. 2:**
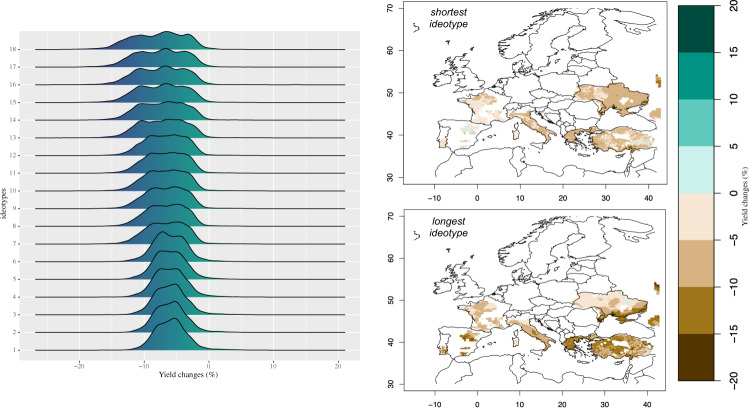
Mean yield changes in 2021-2040 with no adaptation. Left Panel: Spatial probability density function of the ensemble mean yield changes in 2021–2040 (% w.r.t. to 1986–2005) for each ideotype (from the shortest 1 to the longest 18) with no adaptation being implemented. Right panel: Ensemble mean yield changes in 2021–2040 (% w.r.t. 1986–2005) of the shortest and longest ideotypes. Colours in the right panel are associated with the estimated changes.

**Fig. 3:**
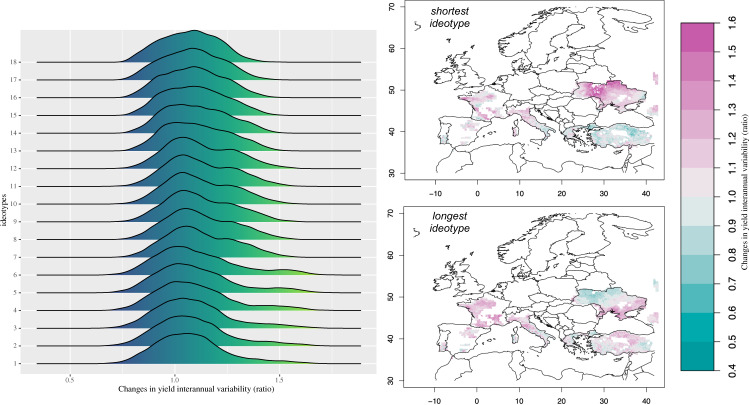
Changes in yield interannual variability in 2021-2040 with no adaptation. Left Panel: Spatial probability density function of the ensemble changes in the yield interannual variability (ratio between 2021–2040 and 1986–2005) for each ideotype (from the shortest 1 to the longest 18). Right panel: Ensemble changes in the yield interannual variability (ratio between 2021–2040 and 1986–2005) of the shortest and longest ideotypes. Colours in the right panel are associated with the estimated changes.

With no climate service in place, yield reductions are estimated for all ideotypes in the near-future. Mean changes from −7.8% to −5.8% (Fig. 2) are estimated despite the CO_2_ fertilisation effect^10^. Spatial differences characterise these losses in many regions. Ideotypes having a shorter growing cycle are projected to be less affected by climate change, with a spatial pattern of yield losses being more homogeneous and having mostly unimodal distribution. While being associated with larger losses, ideotypes with longer growing cycle are also characterised by a more spatially heterogeneous climate change response with distribution mostly tri-modal (Fig. 2). These results point to higher exposure of varieties having longer thermal requirements to reach heading, entering the most critical phenological phases too late to avoid unfavourable conditions and extremes (e.g., drought and spring/early summer heat waves). These spatial differences highlight the need of developing and implementing local adaptation solutions. In the context of this study, they show that a variety being optimal for all producing regions does not exist. Overall, ideotypes with shorter growing cycles achieve better performance in terms of minimising yield losses; however, the targeted use of longer-cycle ideotypes can bring yield benefits in some producing areas. Interannual variability increases from 7% to 12%, under projected climate change conditions and no climate service in place, by following the increase in the growing length of the ideotypes, but it starts decreasing for the ideotypes with the longest growing demand (Fig. 3). This points to the need of adapting considering a trade-off between minimising yield losses and limiting the increase in the interannual variability.

Regional and model-dependent differences characterise the overall picture, especially when looking at varieties minimising the negative effects of climate change in terms of mean yield, interannual variability, or both. These differences are induced by several factors, among them also the higher GCM/RCM inter-model variability in precipitation at near-future temporal scales^24^.

Therefore, a regional approach should be taken to identify the optimal durum wheat ideotype enhancing climate resilience. In Ukraine, for instance, future yield-optimal wheat varieties, minimising the projected mean yield losses, are the ones having a longer growing cycle in two regional climate model (RCM) driven ensembles out of five, while there is one showing a more complex east-west gradient (Fig. S1 in the Supplementary). Similar patterns are identified for northern Italy, where three RCM-driven ensembles of simulations out of five suggest that longer growing cycle varieties are better for mean yield under future climate conditions. Regions having a more homogeneous response to the different RCM-driven ensembles of simulations still show spatial differences worth to be mentioned and understood. This is the case of southern Italy, with future yield-optimal varieties (in terms of mean yield losses) having shorter-to-average growing cycles. This behaviour suggests a higher precipitation-sensitivity in wheat yield response.

Another important aspect that should be considered in designing variety-based adaptation strategies is that future optimal varieties in terms of mean yield losses are not necessarily also the best ones in reducing the negative impacts of climate change on interannual variability (Fig. S2 in the Supplementary). In order to address this issue, we also employed an index of crop yield resilience^25^ (see Methods), accounting at the same time for both mean yield and interannual variability. The response to climate change in terms of resilience shows higher models’ coherency, although not in all regions. In most of Ukraine, for instance, the resilience-optimal wheat varieties (i.e., the ones minimising the projected decrease in resilience) are the ones having the longest growing cycle. A more complex response, however, emerges in the durum-wheat producing areas of France with two RCM-driven ensembles suggesting varieties with an average-to-longer growing cycle as resilient-optimal and other three pointing to shorter-to-average ones (Fig. S3 in the Supplementary).

**Fig. 4:**
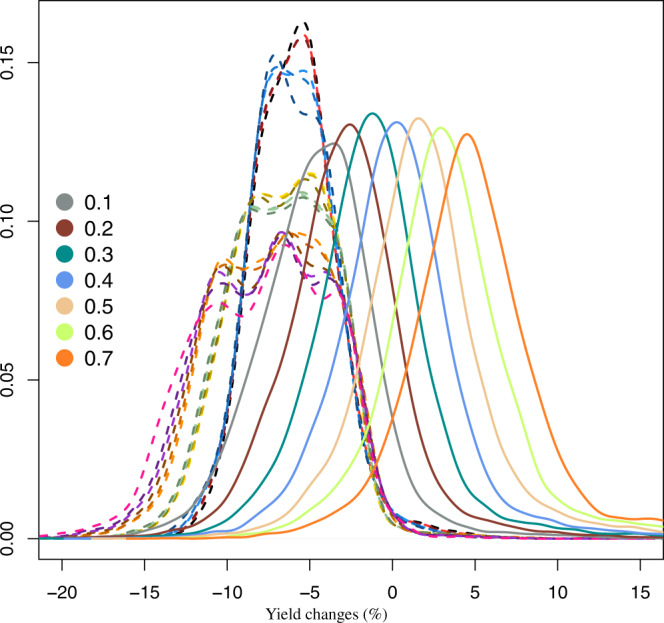
Effects of the idealised climate service on mean yield changes. Spatial probability density functions of the ensemble mean yield changes (%; 2021–2040 vs 1986–2005) estimated for each ideotype (dotted line) and for the simulations integrating the idealised climate service with different levels of skill (bold lines and according to the colours from 10% to 70%).

Positive effects, highlighting the key role of adaptation, are obtained by integrating the idealised climate service (Fig. 4) informing at sowing on varieties to be sown. This service supports year-by-year (in the projected future time period) the choice of varieties to be sown with a set of (not evolving in time) a priori imposed prediction skills (see Methods). The ensemble of simulations integrating the climate service outperforms all previous results in terms of effects on mean yield. Moreover, when the prediction skill reaches 40%, the negative impacts of climate change on mean yield are offset; while for higher skill, mean yield gains are projected under future climate conditions (reaching a value of 5.3% w.r.t. the baseline with a prediction skill of 70%). The positive effects on mean yield induced by climate services having lower skill points to the role of variety mixtures^26^ (although over time and not in space here) in reducing the negative impacts of climate change.

**Fig. 5:**
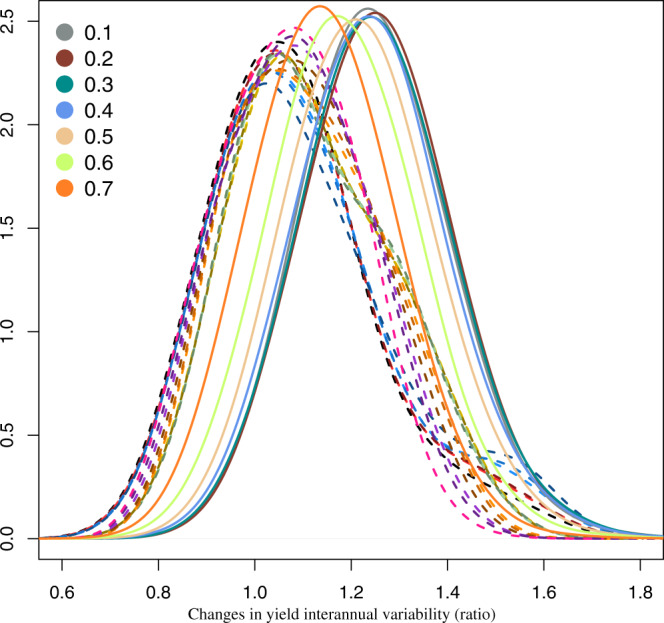
Effects of the idealised climate service on changes in yield interannual variability. Spatial probability density functions of the ensemble changes in the yield interannual variability (ratio 2021–2040, 1986–2005) estimated for each ideotype (dotted line) and for the simulations integrating the idealised climate service with different skill (bold lines and according to the colours from 10% to 70%).

**Fig. 6:**
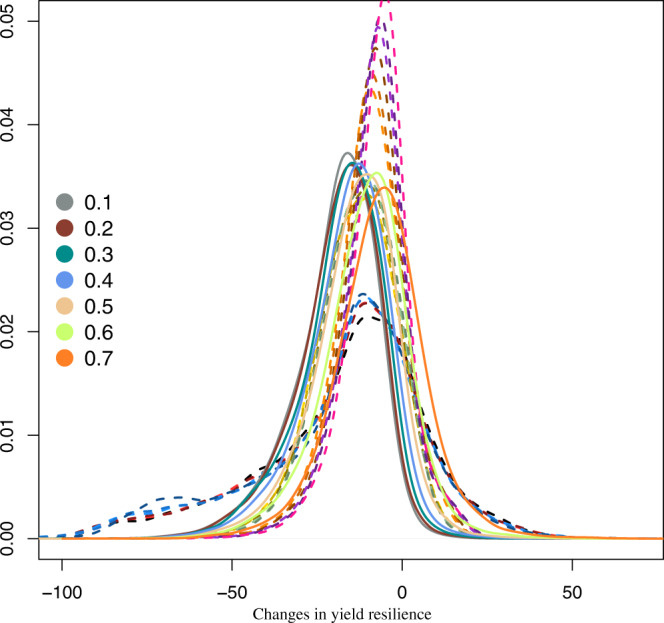
Idealised climate service and changes in resilience. Spatial probability density functions of the ensemble resilience changes (2021–2040 minus 1986–2005) estimated for each ideotype (dotted line) and for the simulations integrating the idealised climate service with different skill (bold lines and according to the colours from 10% to 70%).

Unfortunately, these positive results in terms of mean yield come along with an increase in interannual variability oscillating around 25% for climate services having prediction skill lower than 50% (Fig. 5). At higher prediction skill, results reveal a much lower increase in the interannual variability while achieving remarkable yield gains. The same patterns is revealed when considering resilience indicators. Despite the positive results in terms of mean yield response, only climate services informing on crop variety with a skill at least of 70% achieve an overall trade-off balance (Fig. 6).

## Discussion

Estimating the impacts of climate change in complex sectors (like agriculture), highly influenced by socio-economic and local human decisions, and the effects of plausible adaptation strategies is still challenging. Differences emerge with respect to varieties minimising mean yield losses and reducing the impacts on yield interannual variability assuming no future use of climate services. To fully capture the complexity of these impacts and the effects of different agro-management decisions, there is a need to employ very large (in the order of hundreds) ensemble of climate-crop models’ simulations extending our approach and results. For instance, here the ideotypes (used to simulate durum wheat varieties a farmer may have access at) have been built by focusing on a single-trait. Other important traits, and the combination of them, should be explored. Specific models’ sensitivities^27^ to different parameters to be calibrated should be also considered in developing very large ensembles.

In agriculture, adapting through dynamic variety selection at sowing is amongst the most likely strategy to be implemented due to its high benefit-to-cost ratio. Integrating a climate service supporting variety selection at sowing definitely ameliorates the impacts of climate change in terms of mean yield changes of durum wheat in the Euro-Mediterranean region. However, its applicability may be limited by the associated increase in the interannual variability (that may trigger price volatility and market instability). This is, however, not valid for climate services informing on durum wheat variety to be sown with prediction skill equal (or greater than) 70%. As many end-users have been advocating during last years in the co-design process of several European climate services, a skill of 70% seems indeed to represent a watershed. Lower value may still be acceptable if dedicated stabilisation mechanisms will be developed and applied to avoid higher interannual yield variability triggering higher market volatility, and to further support famers’ income stability.

The beneficial effects of climate services with lower skill also seem to support the key role that variety mixtures may play under future climate change conditions. Despite the mixture being realised in time here (rather than in space, and as a side product of climate service advising farmers on variety to be sown), useful lessons may be learnt to start building optimised mixtures^26^.

While confirming the negative impacts of climate change, our findings show how targeted dynamic adaptation strategies (realised by using a sectoral and tailored climate service) can help to reduce yield losses and in some cases also turn them into gains. Our results contribute to further support the use of sectoral climate services as an effective adaptation tool. Although, there is still a long way to reach the desired accuracy that will make them fully profitable. Besides crop and crop variety choices, many other key farm’s decisions and actions can be supported by climate services^22^. Analysing the effectiveness of climate services with respect to the full set of possible actions still represents a challenge that may be addressed (in the near future) by innovative integrated farm system modelling approaches^28^.

Our findings also highlight the need of a dynamic approach to adaptation. An effective and sustainable adaptation strategy cannot be designed today and applied as it is for the coming decades. There is a need for a dynamic approach, closely involving end-users, based on constant monitoring, feedback, re-design, and re-implementation.

## Methods

The five regional climate models here used come from the EURO-CORDEX initiative^29^ and were run under the RCP8.5 scenario from 2006 on at 0.11 degree of spatial resolution (Table S1 in the Supplementary). Daily maximum and minimum temperatures, total precipitation were bias-adjusted by using quantile mapping, while a model-by-model evaluation was performed for global solar radiation^2^. The crop growth model here applied is ECroPS, the new model developed by the European Commission Joint Research Centre. ECroPS builds on the WOFOST model^30,31^. As for the effects of climate and climate extremes, the model simulates the impacts of water limitation, heat stress effects at flowering, and heat stress effects during grain filling. ECroPS also takes into account the so-called CO_2_ fertilisation effects^10^. The simulations do not take into account nutrient limitations and the effect of pest and diseases. Soil data, calibrated parameters, crop calendar, etc. of ECroPS have been all derived by the ones in place at the European Commission Joint Research Centre for the MARS crop monitoring and forecasting activity^32,33^.

The 18 ideotypes were built by using 191 accessions^19,23^ collected from experiments in the Mediterranean region and chosen to represent the genetic diversity of durum wheat. Nine field experiments in 4 different countries of the Mediterranean region were used to build this dataset^23^. The 18 ideotypes here used have been extracted by random sampling (with no replacement) from the three components of the estimated multi-modal distribution characterising the heading thermal requirements of the accessions dataset. Their thermal requirements to reach both flowering and maturity were then used to run ECroPS. The ideotype with the shortest growing cycle reaches flowering with 1040 degree days^34^ (base temperature at 0 °C), while the longest one needs 1507 degree days (Table S2 in the Supplementary). Sowing date was kept fixed in each simulated year among the ideotypes to avoid dealing with another confounding factor, while its grid-dependent value was retrieved from the EU-JRC MARS operational crop monitoring and forecasting system^32,33^. The number of the ideotypes represents a compromise between the computational demand of the simulations and the need of sampling the three identified families of the estimated multimodal distributions.

Differences in the mean crop yield response to the different climate conditions (2021-2040 vs 1986-2005) are assessed by using a 2-sample Anderson-Darling test. The resilience index here applied is defined^25^ as ${\mu }^{2}/{\sigma }^{2}$, with *μ* and $\sigma$ being the mean and the standard deviation of the simulated crop yield, respectively. The idealised climate service, informing on wheat variety to be sown each year of the projected time period, was implemented in a post-processing phase on the full set of simulations done for the 18 ideotypes (Fig. S4 in the Supplementary). The idealised climate service indeed aims at reproducing a real one based on seasonal climate predictions, local information, and phenological modelling^20^. For each simulated year, the *reality* (given by the crop growth model forced by the climate projections) is supposed to be known. Thus, an idealised service can be realised by applying a probability of success in selecting the (known) optimal variety (i.e., the one achieving the highest yield) from the pool of simulated ones. At each grid point, the time series of projected yield, obtained by employing the idealised climate service, is then built by mimicking the service through a Boolean Bernoulli approach. Thus, with probability *p*, the best variety (i.e., the one having the highest simulated yield for that projected year among the 18) is chosen at each grid point. The probability of success (i.e., selecting the variety achieving the highest yield among the 18 ideotypes) *p* is given by the prediction skill of the idealised climate service, expressed as hit rate characterising the joint probability of correctly predicting and observing an event. Clearly, this is a simplified approach that does not account for spatial and temporal dependencies, e.g., a farmer may be influenced by what is chosen by closer farmers and/or yield performance obtained in the past. Furthermore, this approach assumes farmers not having any variety restriction (that may arise from specific contracts) and being able to access every year a representative pool of durum wheat varieties. Finally, to build the ensembles integrating the idealised climate services, at fixed hit rate (prediction skill in the main text), we used all regional climate models as well as all baseline runs with each of the 18 ideotypes for each grid-point (assuming no climate service was in place and considering that no precise information of specific varieties is available at the gridded scale of the simulations).

### Reporting summary

Further information on research design is available in the Nature Portfolio Reporting Summary linked to this article.

## Supplementary information

Supplementary Information

Reporting Summary

## Data Availability

The full wheat accession data are available by contacting C.R. The thermal requirements of all varieties are available by contacting A.T. The bias-adjusted regional climate model simulations can be retrieved at the European Commission Joint Research Centre Data catalogue https://data.jrc.ec.europa.eu/. The wheat simulated data are available by contacting A.T. or at the EC-JRC Agri4cast Data portal https://agri4cast.jrc.ec.europa.eu/DataPortal. Wheat projections for all the 18 ideotypes can be further explored at https://ec-jrc.shinyapps.io/medgold/.

## Code availability

The ECroPS model (implemented in python) is available at https://github.com/ec-jrc/ecrops. All the other procedures have been implemented in R and can be obtained by contacting A.T.
