# Supplementary material for: Climate service driven adaptation may alleviate the impacts of climate change in agriculture
Source: Commun Biol. 2022 Nov 12;5:1235. doi: 10.1038/s42003-022-04189-9 (PMC9653372; doi:10.1038/s42003-022-04189-9)
Supplement: Supplementary file 1 — Supplementary Information [file 42003_2022_4189_MOESM1_ESM.pdf]

Supplementary Material

Climate service driven adaptation may alleviate the impacts of climate change in agriculture

Andrea Toreti[1], Simona Bassu[1], Senthold Asseng[2], Matteo Zampieri[1,4], Andrej Ceglar[1,5], Conxita Royo[3]*

1 European Commission, Joint Research Centre, Ispra, Italy

2 Technical University of Munich, Freising, Germany

3 IRTA, Lleida, Spain

4 Present address: King Abdullah University of Science and Technology, Thuwal, Kingdom of Saudi Arabia

5 Present address: Climate Change Centre, European Central Bank, Frankfurt, Germany

** Corresponding author: andrea.toreti@ec.europa.eu*

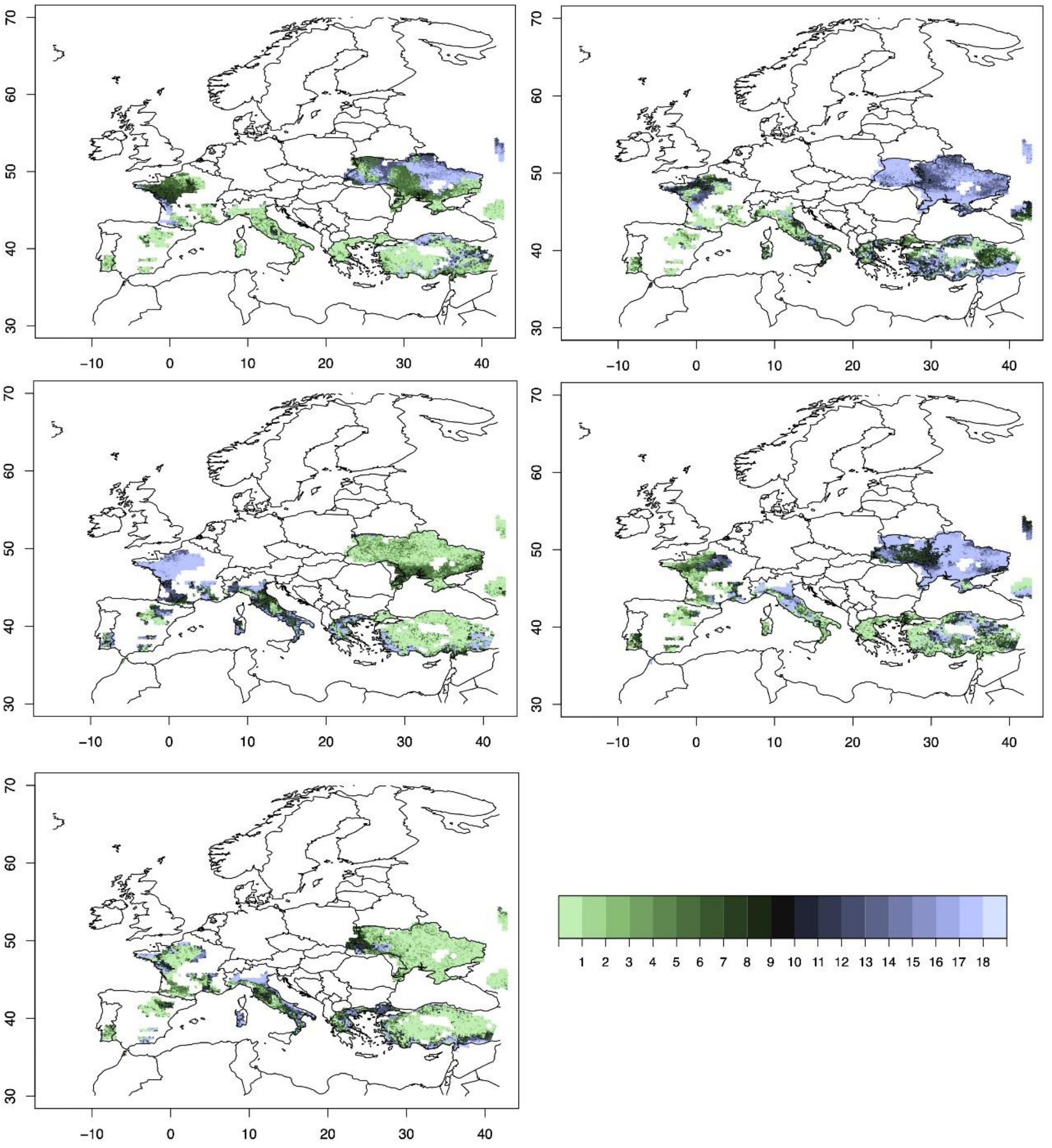

Figure S1. Estimated ideotypes minimizing mean yield losses for each of the 5 analysed RCMs under the RCP8.5 emission scenario in 2021-2040.

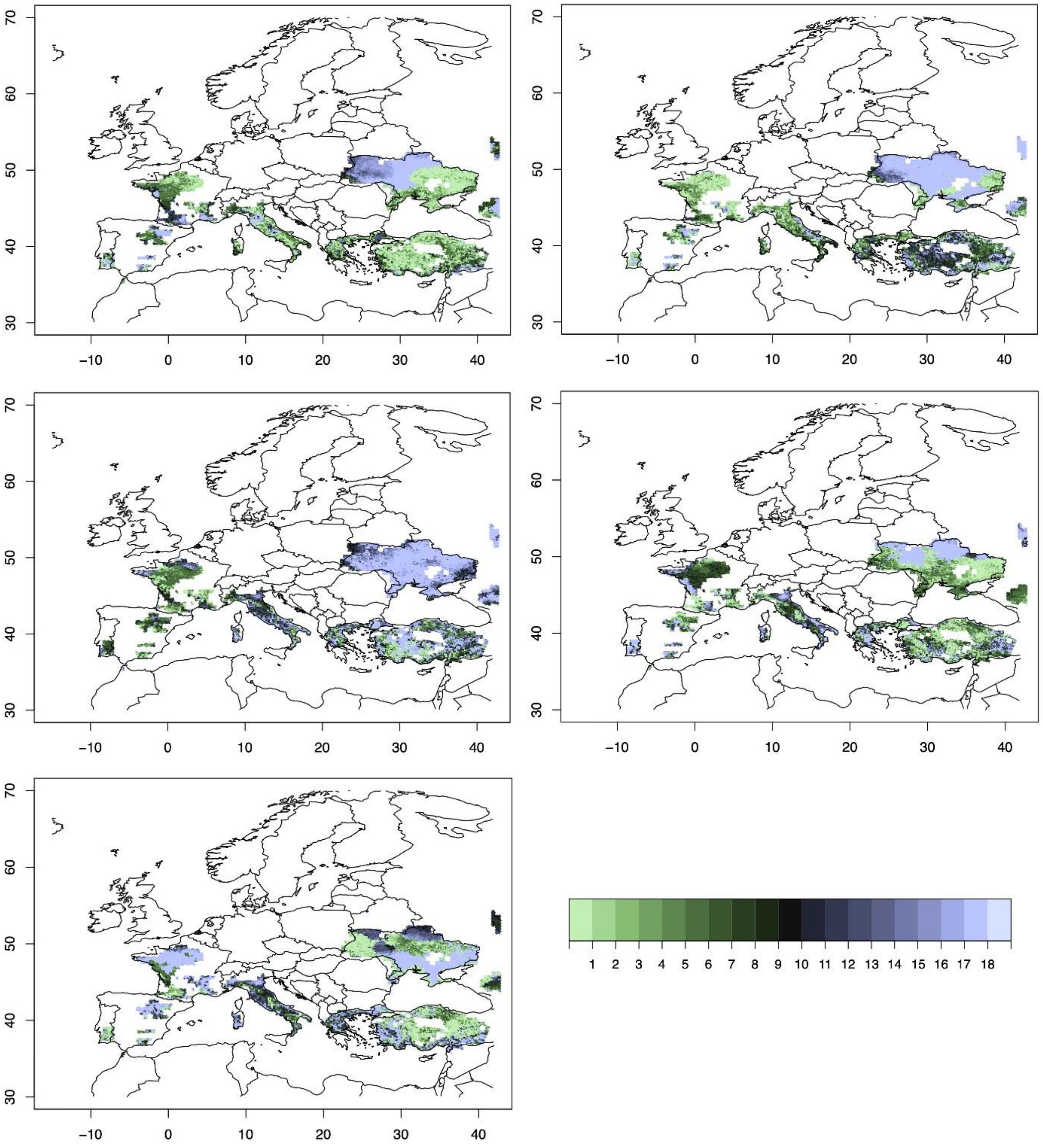

Figure S2. Estimated ideotypes minimizing yield interannual variability for each of the 5 analysed RCMs under the RCP8.5 emission scenario in 2021-2040.

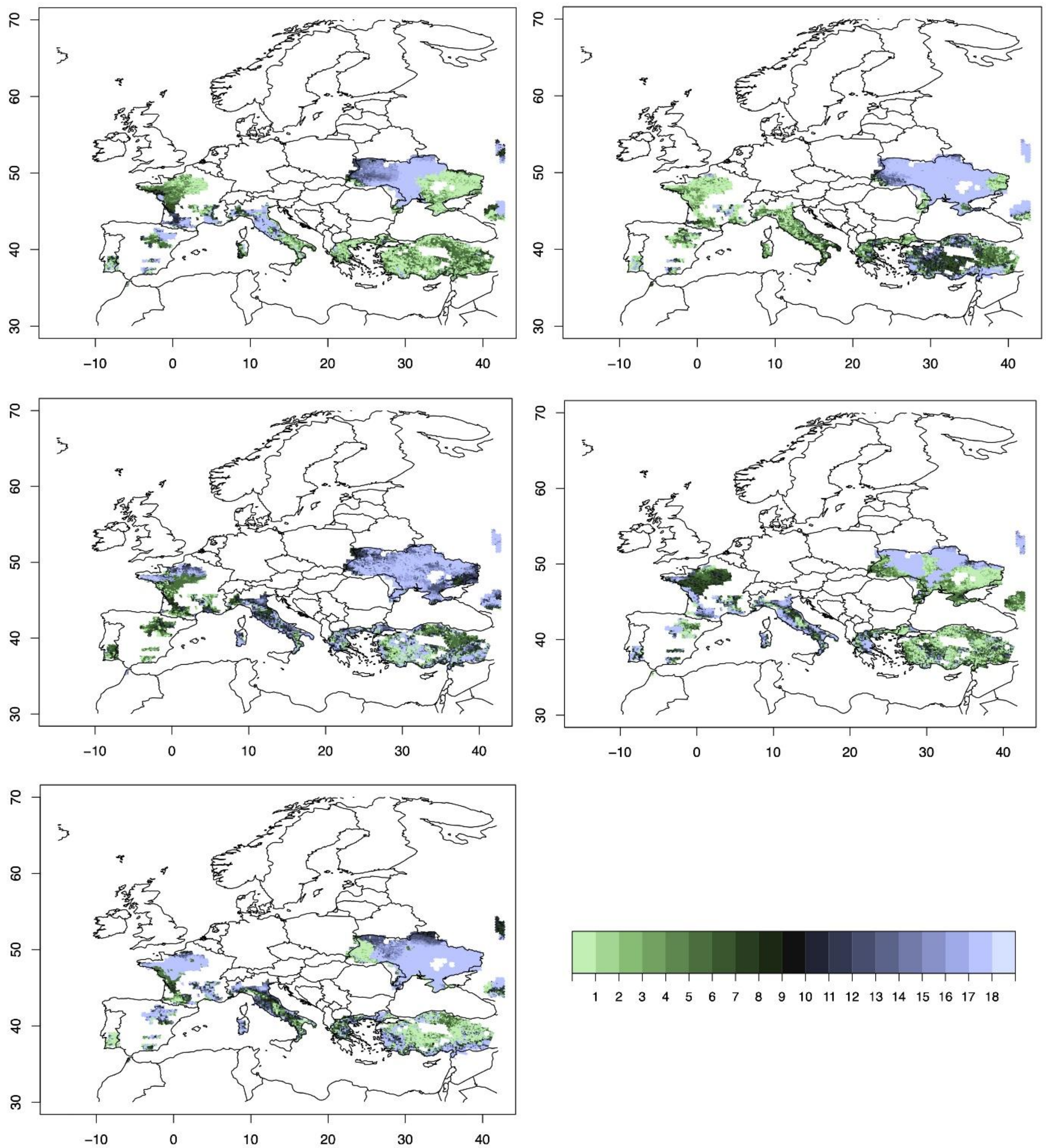

Figure S3. Estimated ideotypes minimizing resilience losses for each of the 5 analysed RCMs under the RCP8.5 emission scenario in 2021-2040.

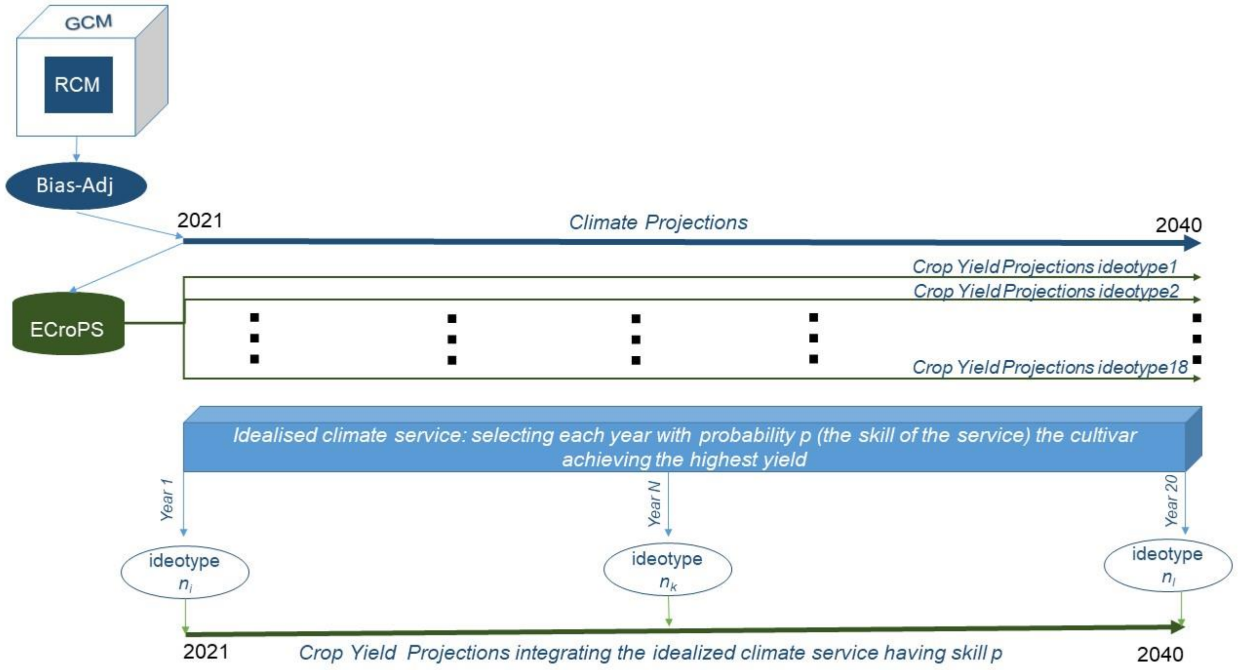

Figure S4. Schematic representation of the approach here developed and implemented to estimate climate change impacts and to mimic the integration of an agro-climate service informing (with a time-independent skill p a priori-set) on varieties to be sown based on seasonal climate forecast, local information, and phenological modelling.

Table S1. The 5 Euro-CORDEX GCMs-RCMs combinations used in this study

RCM	***GCM***
CCLM4-8-17	*CNRM-CERFACS-CNRM-CM5*
CCLM4-8-17	*ICHEC-EC-EARTH*
WRF331F	*IPSL-IPSL-CM5A-MR*
RCA4	*MOHC-HadGEM2-ES*
RCA4	*MPI-M-MPI-ESM-LR*

Table S2. Thermal anthesis requirement (expressed in degree days) of the 18 ideotypes from the 3 identified families

Ideotype 1	*1040*
Ideotype 2	*1052*
Ideotype 3	*1054*
Ideotype 4	*1069*
Ideotype 5	*1073*
Ideotype 6	*1091*
Ideotype 7	*1292*
Ideotype 8	*1295*
Ideotype 9	*1304*
Ideotype 10	*1313*
Ideotype 11	*1315*
Ideotype 12	*1321*
Ideotype 13	*1400*
Ideotype 14	*1410*
Ideotype 15	*1431*
Ideotype 16	*1449*
Ideotype 17	*1464*
Ideotype 18	*1507*
